# Deterministic Capture of Individual Circulating Tumor Cells Using a Flow-Restricted Microfluidic Trap Array

**DOI:** 10.3390/mi9030106

**Published:** 2018-03-02

**Authors:** Yousang Yoon, Jusin Lee, Ki-Chun Yoo, Onejae Sul, Su-Jae Lee, Seung-Beck Lee

**Affiliations:** 1 Department of Electronic Engineering, Hanyang Universtiy College of Engineering, 222 Wangsimni-ro, Seongdong-gu, Seoul 04763, Korea; ysyoon88@hanyang.ac.kr (Y.Y.); jusin19@hanyang.ac.kr (J.L.); 2 Department of Life Science and Research Institute for Natural Sciences, Hanyang Universtiy College of Natural Sciences, 222 Wangsimni-ro, Seongdong-gu, Seoul 04763, Korea; vanity0706@gmail.com (K.-C.Y.); sj0420@hanyang.ac.kr (S.-J.L.); 3 Institute of Nano Science and Technology, Hanyang Universtiy, 222 Wangsimni-ro, Seongdong-gu, Seoul 04763, Korea; ojsul@hanyang.ac.kr

**Keywords:** circulating tumor cell, single-cell capture, microfluidics

## Abstract

Circulating tumor cells (CTCs) are regarded as a strong biomarker which includes clinically valuable information. However, CTCs are very rare and require precise separation and detection for effective clinical applications. Furthermore, downstream analysis has become necessary to identify the distinct sub-population of CTCs that causes metastasis. Here, we report a flow-restricted microfluidic trap array capable of deterministic single-cell capture of CTCs. The extent of flow restriction, correlating with the device geometry, was then optimized using a highly invasive breast cancer cell line (LM2 MDA-MB-231) to achieve 97% capture efficiency with a single-cell capture rate of 99%. Single-cell capture of CTCs from mice with full-blown metastasis was also demonstrated. The single-CTC capturing ability of the flow-restricted trap array not only showed cell enumerating ability but also high prospects for application in future automated downstream analysis.

## 1. Introduction

It is well known that the main cause of cancer metastasis is circulating tumor cells (CTCs) which are derived from the primary tumor site, circulate in the bloodstream, relocate in distant organs and then create tumors at secondary sites. Researchers have reported that CTCs could be found in early stages of carcinogenesis even before the emergence of clinical symptoms [1,2], and the number of CTCs correlates closely with patient survival rates [3,4]. Therefore, CTCs can perform as a strong biomarker with clinically valid information which can be applied in early cancer diagnosis, prognosis, and therapeutic assessment.

However, CTCs are very rare in patients’ blood: just a few to less than 10 CTCs per 10^9^ hemocytes [5,6]. Therefore, to fulfil the high clinical potential of CTCs, techniques that precisely separate and detect CTCs at extremely low concentrations are required. A typical approach to capture and identify CTCs is by utilizing the tumor-specific antibodies as reflected by the epithelial cell adhesion molecule (EpCAM). However, heterogeneity of epithelial molecules among cancer types and even from the identical origin results in limited efficiency and biased information [7,8]. An alternative approach to capturing CTCs is to utilize the distinct physical property of CTCs, suggesting that CTCs are generally larger and more rigid than the other blood cells. Typically, size-based filtration systems have been utilized since they show reasonable capture efficiency with high potential for automation while being simple and robust [9,10,11,12,13]. However, even well-defined filters get easily disturbed by an accumulation of captured cells leading to decreased pore size and number of trap sites. Consequently, smaller non-target cells and debris may surround the captured CTCs, disturbing CTC detection and degrading capture purity, and, even worse, the cell accumulation may clog the filters and raise the hydraulic pressure, causing loss of CTCs.

Various techniques have been introduced to overcome the limitations in both immunocapture and size-based filtration methods [14,15,16,17,18]. However, even successful systems overlook the need for single-cell capture of CTCs. Captured cells were randomly distributed and some were lumped together, which makes the enumeration and analysis of captured CTCs a labor-intensive process. Furthermore, recent studies have revealed that there exists a genetic discordance between a primary tumor and CTCs [19,20], and a distinct sub-population among CTCs which had undergone migratory epithelial-to-mesenchymal transition (EMT) of phenotype [1,2,5,8]. These results imply the necessity of single-cell capture of viable CTCs that enables users to identify the captured cells individually and analyze the distinct subset of interest along with precise enumeration.

Considering the scarcity of CTCs, single cell seeding into well plates by serial dilution or droplet microfluidics may not be the optimal candidate. Both the capture efficiency and the single-cell capture rate must be remarkably high to derive valuable information for clinical applications. Single bead trapping by geometrically-induced flow resistance manipulation reported by Tan and Takeuchi pioneered a competitive method for single-cell capture [21] followed by advanced applications for single-cell manipulation and analysis [22,23,24,25,26,27,28]. However, mostly using the well-defined distinct size of cells, few were accessible for capturing CTCs which require varied sizes of cells to be processed.

Here, adopting the principle, we report a microfluidic flow-restricted trapping array device for capturing CTCs with an improved single-cell capture rate. The flow restriction structure at the trapping channel was applied to achieve single-cell capture without channel clogging. To verify our argument, we compared a laterally restricted (LR) geometry with a further vertically restricted (fVR) geometry using computational analysis, experimentally analyzed using polystyrene (PS) particles, and a highly invasive breast cancer cell line (LM2 MDA-MB-231). By correlating the results, we were able to optimize the flow-restricted trapping array that achieved high capture efficiency and single-cell capture rates, allowing the demonstration of the single-cell capture of CTCs from mice whole blood.

## 2. Materials and Methods

### 2.1. Concept

**Figure 1:**
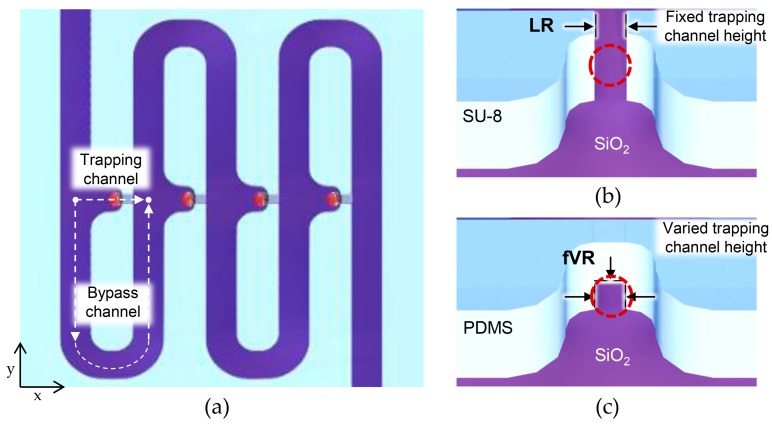
Schematic images of the deterministic cell capture: (**a**) schematic top view of the microfluidic device; (**b**) schematic illustrations of capture sites of the laterally restricted (LR)-type and (**c**) further vertically restricted (fVR)-type.

Figure 1 shows the geometry of the flow-restricted single-cell capture array. Every cell capture site had two paths: a trapping channel, and a bypass channel to the next capture site. To ensure cell flow toward the capture site, flow resistance to the trapping channel was designed to be smaller than that of the bypass channel by stretching the bypass channel length accordingly (0.4–2.4 mm; volumetric flow rate ratio (Q trap/Q bypass) being 1.7). Once the capture site was occupied, its flow resistance became relatively higher, guiding the approaching cell to the next capture site. To ensure single-cell capture for every capture site, a target cell must block the trapping channel enough to inhibit further fluid flow that may guide another cell to be captured. When the flow restriction at the capture site was realized using a single lithography step, there was a limitation in reducing the cross-sectional area of the trapping channel since the channel height should be high enough to accommodate varied sizes of tumor cells while preventing device clogging. Therefore, most tumor cells could not block the trapping channel enough, allowing further fluid flow that may lead to multiple cell capture (Figure 1b). The fVR trapping array, on the other hand, was able to reduce the trapping channel dimension enough to limit further fluid flow, reducing multiple-cell capture (Figure 1c).

### 2.2. Computational Analysis

**Figure 2:**
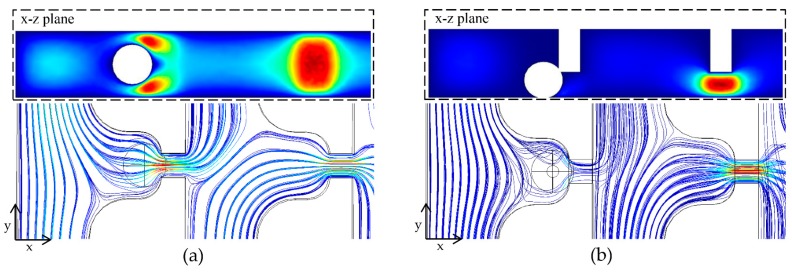

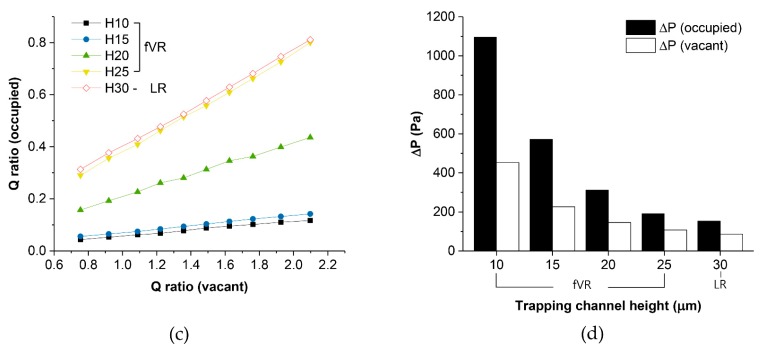
Computational analysis of the deterministic cell capture: (**a**) velocity field and streamlines of an LR-type and (**b**) an fVR-type; (**c**) Q ratio of the occupied trap sites regarding the Q ratio of the vacant trap sites for various trapping channel heights; (**d**) pressure difference across the occupied and vacant trapping channels for various trapping channel heights.

The fluid flow through two consecutive capture sites with varying height of the trapping channels was studied using COMSOL™ 5.2 (COMSOL, Inc., Stockholm, Sweden) to study the hydrodynamic conditions and correlate with the device performance. Figure 2a,b shows the velocity field and streamlines of the LR-type referring to the trapping channel height of 30 μm and the fVR-type referring to the trapping channel height of 10 μm. The preceding capture sites were designed to be occupied by the spheres 18μm in diameter. It can be seen that the number of streamlines to the occupied capture site were drastically decreased in the fVR-type compared to the LR-type, indicating less possibility of multi-cell capture. The number of streamlines refers to the volumetric flow rates (Q) which could be calculated by surface integration of the cross-section, and the relationship between the Q ratio (Q trap/Q bypass) of the occupied and the vacant sites for a trapping channel height of 10 to 30 μm was numerically analyzed (Figure 2c). As can be seen, the Q ratio of the occupied capture site was proportional to that of the vacant capture site; however, its slope depended on the trapping channel height forming three groups compared to the captured particle size. Furthermore, the pressure difference across the capture site was also analyzed considering that cells could be deformed and escape through the trapping channel depending on the applied hydraulic pressure. With the Q ratio at the vacant site and the flow rate from the inlet being constant, the pressure differences of both occupied and vacant capture sites exponentially increased as the trapping channel height decreased (Figure 2d).

### 2.3. Fabrication Method

**Figure 3:**
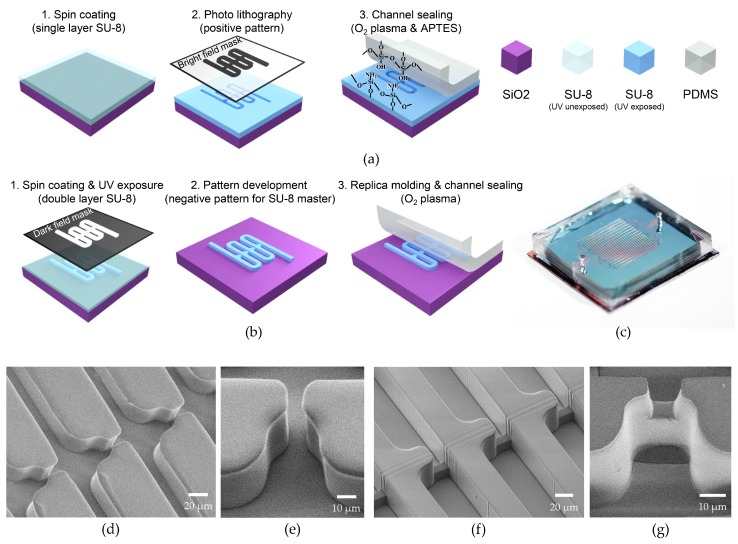
Fabrication of flow-restricted microfluidic trap array: (**a**) fabrication process of (**a**) LR-type and (**b**) fVR-type devices; (**c**) optical image of a flow-restricted microfluidic trap array; (**d**) SEM image of an LR-type device and (**e**) its capture site; (**f**) SEM image of an SU-8 master for an fVR-type device and (**g**) its poly(dimethylsiloxane) (PDMS) molded capture site.

Both LR-type and fVR-type microfluidic devices were realized on oxidized chips with a 2 × 2 cm^2^ surface. The LR-type microfluidic channel was patterned by photolithography using SU-8 2050 (Microchem, Westborough, MA, USA) negative photoresist which was spin-coated to be 30 μm thick. Bright field mask was utilized for the positive patterning of SU-8 2050 which became the channel wall. For the channel encapsulation, 5-mm thick poly(dimethylsiloxane) (PDMS) was used with inlets and outlets defined. To firmly seal the device, patterned SU-8 was exposed to O_2_ plasma (35 W, 100 mTorr, 30 s) followed by dipping in 5% (3-Aminopropyl)triethoxysilane for 10 minutes at 80 °C. Then it was attached to the PDMS that was treated with O_2_ plasma (35 W, 100 mTorr, 30 s) (see Figure 3a).

The fVR-type microfluidic devices were fabricated using soft-lithography. The fabrication process of the SU-8 masters for the trapping channel height of 10 μm and 15 μm is as follows. Ten micrometer-wide straight lines which defined the trapping channels were UV-exposed on SU-8 2010 (Microchem) using a dark field mask. Then, without the development process, SU-8 2050 was spin coated and the bypass channel patterns were UV-exposed followed by the development of the whole microfluidic channel. For the trapping channel height of 20 μm, on the other hand, the types of SU-8 for the first layer and the second layer had to be exchanged, and the pattern for the first layer had to include the bypass channels, requiring more precise pattern alignment between the layers. This was due to the higher solvent content reducing the height of the unexposed area of the first SU-8 layer (see Figure 3b). The fabricated SU-8 master was placed on a Petri dish, and the degassed PDMS was poured followed by curing at 80 °C in an oven for 30 min. The PDMS was cut and detached from the SU-8 master followed by punching holes for the inlet and the outlet. The PDMS microfluidic channel was attached on an oxidized Si chip with both surfaces treated by O_2_ plasma (35 W, 100 mTorr, 30 s). Both the SU-8 and PDMS channel walls of the LR- and fVR-type microfluidic devices showed similar hydrophobic properties which did not show differences in fluid flow behavior.

Figure 3c shows an optical image of the fabricated flow-restricted trapping array. It had 8 to 16 parallel microfluidic channels depending on the bypass channel length, and each channel had 130 capture sites. The cross-section of the bypass channel was set to be 30 μm × 30 μm which was the minimum area obtained from previous experiments that was needed to avoid channel clogging, and the width and length of the trapping channels were set to be 10 μm. Scanning electron microscope (SEM) images of the laterally restricted channel are shown in Figure 3d,e. The 30 μm SU-8 2050 forms the fluidic channel walls with a 10 μm lateral restriction at the capture site. Figure 3f shows the SU-8 master of the fVR trapping array and the molded PDMS fluidic channel is shown in Figure 3g. Slight deviation of the restriction gap width originates from the resist profile of SU-8 2010 which formed a trapezoid shape. This was transferred to the molded PDMS restriction channel resulting in widening at the base, by a few microns.

### 2.4. Sample Preparation

Polymer particles: Polystyrene (PS) microparticles (Sigma-Aldrich, St. Louis, MO, USA), 20 μm in diameter, were chosen. The PS particles were suspended in a 15% sucrose solution to expand the time of sinking during the experiments, and surfactant, 0.1% F-127 (Sigma-Aldrich), was also included to disperse the microspheres preventing aggregation.

Tumor cells: Human breast cancer cell line MDA-MB-231 was purchased from American Type Culture Collection (Manassas, VA, USA). The cells were maintained at 37 °C with 5% CO_2_ in Dulbecco’s modified Eagle medium (DMEM) supplemented with 10% fetal bovine serum (FBS), penicillin (100 U/mL), and streptomycin (100 μg/mL). By tail vein injection into athymic nude mice, MDA-MB-231 cells were metastasized to the lung and the lung-metastasized MDA-MB-231 cells were named LM1. Likewise, LM1 MDA-MB-231 cells were injected into the mouse tail vein and metastasized again to the lung. Because those cells were metastasized twice to the lung organ, they were designated as LM2 MDA-MB-231 cells.

Animal experiments: MDA-MB-231 (1 × 10^6^) cells that were transduced with green fluorescent protein (MSCV-IRES-eGFP) were injected into the tail vein of athymic nude mice (n = 4; Orient). After the mice were euthanized with CO_2_ gas, whole lungs were surgically taken and fixed with 4% paraformaldehyde. Lung metastasis was analyzed by counting the number of foci on the lung surface by the naked eye under a microscope. This study was reviewed and approved by the Institutional Animal Care and Use Committee (IACUC) of the Center for Laboratory Animal Sciences, Medical Research Coordinating Center, HYU Industry-University Cooperation Foundation.

## 3. Results and Discussion

### 3.1. Demonstration of Deterministic Single-Particle Capture

**Figure 4:**
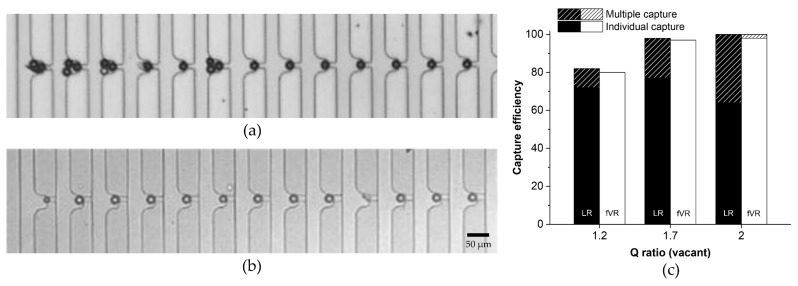
Demonstration of the deterministic polystyrene (PS) particle capture: (**a**) optical image of the particle capture for an LR-type and (**b**) an fVR-type device; (**c**) capture efficiency of the LR- and fVR-type devices regarding various Q ratios. The graph also shows the individual capture rate.

PS particle suspension of ~2 × 10^3^ cells/mL was injected using a syringe pump (Chemyx, Stafford, TX, USA) with the flow rate of each channel at 50 μL/h. The sample was injected until all of the capture sites were occupied. Figure 4a,b shows the captured PS particles in the LR- and fVR-types (trapping channel height of 10 μm). Among the first 13 capture sites, four had multiple PS particles captured in the LR-type (Figure 4a) while no multiple capture was observed in the fVR-type (Figure 4b). Even the smaller particles and debris were individually captured in the fVR-type device. Figure 4c shows the capture efficiency and the individual capture rate of the two types of devices for the Q ratios (vacant) of 1.2, 1.7 and 2. The capture efficiency was evaluated as the number of the occupied capture sites divided by the total number of processed capture sites. High capture efficiency (>98%) was achieved for the Q ratio (vacant) over 1.7, and the capture efficiencies of the two types showed little difference for all Q ratios (vacant). However, the multiple capture rate was much lower in the fVR-type (<2%) than the LR-type (<36%). This must be due to the insufficient trapping channel blockage during the particle capture for the LR-type, which corresponds to the simulation result.

### 3.2. Demonstration of Deterministic Single-Cell Capture

**Figure 5:**
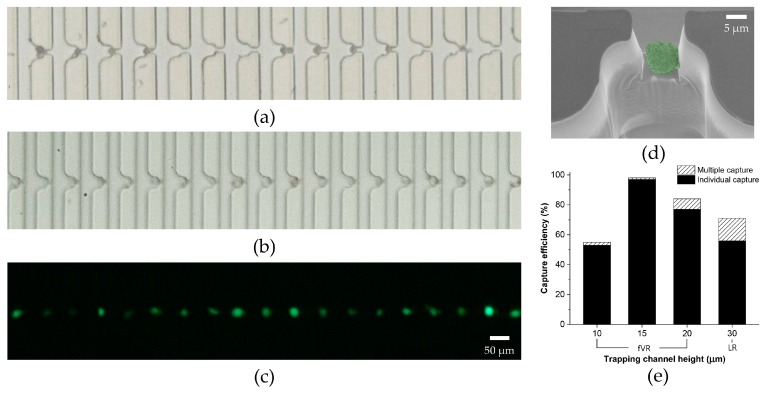
Demonstration of the deterministic cell capture using LM2 MDA-MB-231: (**a**) bright field image of the tumor cell capture using an LR-type device; (**b**) bright field image and (**c**) fluorescent image of the individual tumor cell capture using an fVR-type device; (**d**) SEM image of a captured tumor cell from the fVR-type device; (**e**) capture efficiency of the LR- and fVR-type devices regarding various trapping channel heights. The graph also includes the individual capture rate.

Following the experimental results using PS particles, the device geometries for both types were confirmed to have a Q ratio (vacant) of 1.7. For the tumor cell experiments, the microfluidic device was filled with 0.2% Pluronic F-127 for 15 minutes prior to the sample injection to prevent non-specific adsorption of cells. LM2 MDA-MB-231 cells were observed to have varied diameters from 15–26 μm. Figure 5a–c shows the captured tumor cells in the LR- and fVR-type devices (trapping channel height of 15 μm). In the LR-type device, multiple cell capture and vacant capture sites could be seen between the occupied capture sites. The maximum cell diameter observed among the lost cells was 20 μm. This indicates the inability to accommodate the varied sizes of tumor cells, resulting in low capture efficiency. However, all the capture sites in the fVR-type device shown in the figure were occupied individually by the tumor cells, maintaining both the high capture efficiency and the single-cell capture rate. Figure 5d shows an SEM image of a captured tumor cell in the optimized fVR device. Considering the cell shrinkage caused by the SEM sample preparation process and exposure to a near-vacuum, it can be implied that the size of LM2 MDA-MB-231 cells was larger than the fVR trapping channel cross-section. Figure 5e shows the capture efficiency and the single-cell capture rate for the trapping channel heights of 10 μm, 15 μm, 20 μm, and 30 μm. As the trapping channel height increased from 15 μm to 30 μm, the single-cell capture rate and the capture efficiency decreased. This was due to the higher post-capture cross-sectional area of the trapping channel allowing too much fluid flux. The single-cell capture rate using the trapping channel height of 10 μm was 98%; however, it showed the lowest capture efficiency (55%). This correlates to the simulation result of the pressure difference, inferring that too much vertical reduction can cause excessive hydraulic pressure to the captured tumor cells unless the flow rate is modified accordingly. The excessive pressure can squeeze the captured tumor cell through the trap, leading to cell loss. The optimal capture efficiency of 97% was achieved from the trapping channel height of 15 μm with a single-cell capture rate of 99%. The minimum tumor cell diameter among the captured cells and the maximum diameter among the lost cells were observed as 15 μm. The results indicate that the optimized fVR device, compared to the LR device, was able to achieve a higher single-cell capture rate while accommodating the size variation of LM2 MDA-MB-231 cells.

### 3.3. Single-Cell Capture of CTCs from Mice Whole Blood

**Figure 6:**
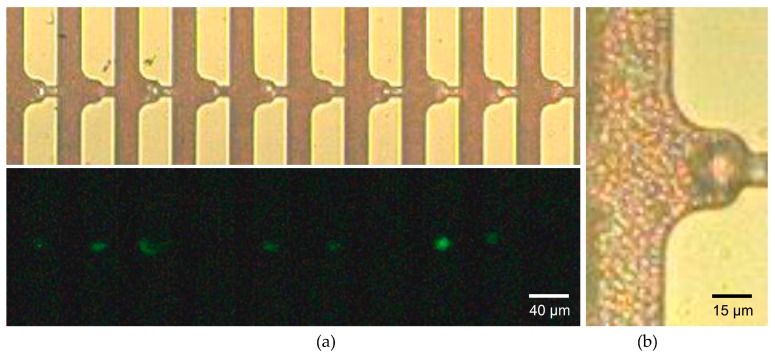
Demonstration of the whole blood processing using fVR trapping array: (**a**) bright field image and its fluorescent image of the individual tumor cell capture using a whole blood sample drawn from the metastasized mice; (**b**) magnified image of a single circulating tumor cell (CTC) capture using a two-fold dilution of the whole blood sample.

Using the optimized device geometry, we proceeded to capture CTCs from whole blood drawn from mice with full-blown cancer metastasis to verify whether the microfluidic device could capture CTCs even with overwhelming numbers of hemocytes. Figure 6a shows the individual capture of the larger cells while the smaller erythrocytes flowed through the bypass and the trapping channels. The initial MDA-MB-231 cells which were injected into the mouse tail vein over-expressed green fluorescent protein. This allowed the captured CTCs to be distinguished from leukocytes or debris by the fluorescent imaging. We evaluated the capture accuracy which was derived as the number of CTCs divided by the total number of occupied capture sites which included leukocytes and debris. A capture accuracy of 71% was achieved, which is reasonably high considering the 10^4^–10^5^-fold abundance of leukocytes compared to the number of CTCs. This may be due to the smaller size and higher deformability of the leukocytes making them more squeezable through the trapping channels. The magnified image of the captured CTC was shown in Figure 6b. We could observe the erythrocytes near the channel wall of the capture site flowing through the trapping channel without being captured. This infers that there exists a narrow flow path for the smaller erythrocytes while inhibiting the larger cells from being captured further, as was shown in Figure 3g. The narrow path might be beneficial for downstream analysis such as immunocytochemistry (ICC), which requires a cell to be exposed to additional solutions.

## 4. Conclusions

We have demonstrated the flow-restricted trap array for the single-cell capture of CTCs from whole blood. Through the computational analysis of fluid flow and experimental verification using PS particles and tumor cells, we were able to optimize the extent of flow restriction and the corresponding device geometry. The optimized flow-restricted trap array achieved a capture efficiency of 97% and a single-cell capture rate of 99% for the LM2 MDA-MB-231 cells. Whole blood drawn from the mice with full-blown metastasis was also applied, achieving a capture accuracy of 71%. The fVR structure allows ICC of the captured cells while being periodically aligned on the same focal plane. This enables a simple detection and enumeration of CTCs by fluorescent imaging. Furthermore, since our device was realized on a silicon chip, there is high potential for integrating various electrical sensors. With the high capture efficiency of the individual CTCs and the accessibility for the downstream analysis, the flow-restricted trap array may bring the automation of CTC detection closer to clinical applications.
